# Palliative Care Awareness Among Caregivers of Cancer Patients in Turkey: A Cross-Sectional Study

**DOI:** 10.3390/curroncol33080458

**Published:** 2026-07-30

**Authors:** Fariz Emrah Özkan, Hacer Demir, Semiha Urvay, Yaşar Culha, Beyza Ünlü, Duygu Özaşkın, Sedat Yıldız, Canan Yıldız, Merve Kuday Özkan

**Affiliations:** 1 Department of Medical Oncology, Afyonkarahisar Health Sciences University, Afyonkarahisar 03030, Türkiye; 2 Department of Medical Oncology, Acıbadem Kayseri Hospital, Kayseri 38140, Türkiye

**Keywords:** awareness, cancer, caregivers, health literacy, palliative care

## Abstract

Background and Objectives: Palliative care awareness among family caregivers plays a critical role in the timely utilization of supportive care services. However, awareness levels and associated factors among caregivers of cancer patients remain insufficiently explored in Türkiye. This study aimed to evaluate palliative care awareness and identify factors associated with awareness among caregivers of cancer patients. Materials and Methods: This cross-sectional study included 550 caregivers of cancer patients receiving treatment at oncology clinics. Sociodemographic characteristics, caregiving-related factors, and palliative care awareness levels were assessed using a structured questionnaire. Associations between awareness and explanatory variables were evaluated using chi-square tests. Independent predictors of awareness were determined by multivariable logistic regression analysis. Results: Among the 550 caregivers included in the study, 277 (50.4%) reported no knowledge of palliative care, 141 (25.6%) reported sufficient knowledge, and 131 (23.8%) reported limited knowledge. Higher educational level, active employment, older age, closer relationship to the patient, advanced disease stage, and previous exposure to palliative care services were significantly associated with greater awareness. In multivariable logistic regression analysis, education level (OR = 2.05, 95% CI: 1.35–3.15, *p* < 0.001), employment status (OR = 1.79, 95% CI: 1.23–2.61, *p* = 0.003), age (OR = 1.04, 95% CI: 1.01–1.07, *p* = 0.004), disease stage (OR = 1.34, 95% CI: 1.05–1.72, *p* = 0.016), caregiver relationship (OR = 1.94, 95% CI: 1.18–3.17, *p* = 0.008), and having a relative who had previously received palliative care (OR = 2.34, 95% CI: 1.42–3.84, *p* < 0.001) were identified as independent predictors of awareness. Conclusions: Palliative care awareness among caregivers of cancer patients remains limited. Educational level, caregiving experience, and exposure to palliative care services significantly influence awareness. Strategies aimed at improving caregiver education and increasing public awareness may facilitate earlier integration of palliative care into oncology practice.

## Simple Summary

Family caregivers are essential partners in the care of people with cancer, yet many have limited knowledge of palliative care and may not recognize its benefits until the advanced stages of disease. Understanding the factors that influence caregiver awareness is important for improving timely access to supportive care services. In this study, we evaluated palliative care awareness among caregivers of patients with cancer and identified the demographic and clinical factors associated with higher awareness. We found that awareness was greater among caregivers with higher educational attainment, employment, previous exposure to palliative care, and those caring for patients with advanced-stage disease. These findings highlight important gaps in caregiver education and emphasize the need for earlier communication about palliative care. Strengthening educational initiatives and integrating palliative care discussions into routine oncology practice may improve caregiver preparedness, facilitate timely referrals, and ultimately enhance the quality of care for patients and their families.

## 1. Introduction

Cancer remains one of the leading causes of morbidity and mortality worldwide and continues to impose a substantial burden on healthcare systems [1]. Beyond its biological and clinical manifestations, cancer represents a complex condition that profoundly affects patients and their families at psychological, social, and economic levels [2]. Therefore, contemporary oncology care increasingly emphasizes not only disease treatment but also the overall well-being and quality of life of patients and their caregivers.

According to the World Health Organization (WHO), palliative care is defined as an approach that improves the quality of life of patients and their families facing life-threatening illness through the prevention and relief of suffering by means of early identification, assessment, and treatment of pain and other physical, psychosocial, and spiritual problems [3]. Importantly, palliative care is not limited to end-of-life care; rather, it should be integrated early in the course of illness and provided alongside disease-directed treatments [4]. The scope of palliative care extends beyond symptom control and includes communication, goal setting, care coordination, and family support [5,6]. In oncology practice, early integration of palliative care has been associated with improvements in quality of life, reductions in anxiety and depression, decreased unnecessary aggressive interventions, and increased patient satisfaction [7,8]. Nevertheless, access to and integration of palliative care services remain inconsistent across healthcare systems and geographical regions [9,10].

One of the major barriers to the timely integration of palliative care is the widespread misunderstanding of the concept among patients, family members, and even healthcare professionals [11]. Palliative care is frequently perceived as a service provided only at the terminal stage of illness or as an indication that curative treatment has been discontinued [12]. Such misconceptions may delay referrals and limit access to interventions that have demonstrated clear benefits for both patients and caregivers. Studies involving patients with advanced cancer and their relatives have shown that awareness of palliative care remains limited and that the concept is often confused with hospice care [13,14]. As a result, palliative care may be perceived as a care option that replaces curative treatment rather than as a complementary approach within the cancer care continuum [11,12].

Cultural and societal factors also play a critical role in shaping perceptions of palliative care. In Turkey, palliative care services have expanded considerably over the past decade through the establishment of hospital-based palliative care units integrated into the national healthcare system. Despite these developments, referral to palliative care often occurs late in the disease trajectory, and public awareness of the scope and benefits of palliative care remains limited. Family members play a central role in caring for patients with cancer in Turkey, making caregiver awareness particularly important for facilitating timely access to supportive care services. In many societies where discussions about death and dying are considered sensitive or taboo, family-centered decision-making patterns tend to dominate [15]. In such settings, physicians may communicate primarily with family members rather than directly with patients regarding prognosis, treatment goals, and palliative care options. Although this approach may be intended to protect the patient, it can inadvertently limit patient autonomy and delay discussions about supportive care strategies [16]. Furthermore, variations in healthcare professionals’ education and training in palliative care may also hinder the early and effective integration of these services into routine oncology practice [5,6]. Strengthening multidisciplinary team approaches, communication skills, and advance care planning practices has therefore been highlighted as an important strategy for improving palliative care delivery [8,10].

Family caregivers play a crucial role in the care of patients with cancer. They often assume multiple responsibilities, including coordinating medical appointments, assisting with symptom management, administering medications, and providing emotional support [2]. These responsibilities may result in significant physical, psychological, and financial burdens for caregivers. The level of knowledge and awareness caregivers possess regarding palliative care may influence not only their own well-being but also the quality of care they provide to patients. Previous studies have shown that misconceptions and limited awareness of palliative care among caregivers can delay access to supportive services and reduce the effectiveness-of-care planning [11,12].

Limited awareness of palliative care may also have broader consequences at the healthcare system level. Health literacy may also influence awareness and acceptance of palliative care. Individuals with limited health literacy may have greater difficulty understanding the objectives of palliative care, distinguishing it from end-of-life care, and navigating available healthcare services. Previous national surveys have shown that health literacy remains suboptimal in a substantial proportion of the Turkish population, suggesting that improving health literacy may represent an important strategy for increasing public awareness of palliative care. Insufficient knowledge about palliative care services has been associated with increased emergency department visits, avoidable hospital admissions, aggressive treatments at the end of life, and higher healthcare expenditures [7,9,10].

Although several international studies have demonstrated that awareness of palliative care among patients with advanced cancer and their caregivers remains low [13,14], most of the available evidence has been derived from Western populations. Cultural norms, family roles, and healthcare delivery systems may differ considerably across regions, potentially influencing perceptions of palliative care and the acceptance of supportive services [15,16]. Therefore, evaluating the level of palliative care awareness among caregivers within specific cultural and healthcare contexts is essential. To our knowledge, this study represents one of the largest cross-sectional investigations of palliative care awareness among caregivers of cancer patients in turkey. In addition to describing awareness levels, it identifies sociodemographic and clinical factors associated with awareness, thereby providing evidence that may support the development of targeted educational strategies and improve timely integration of palliative care services.

In this context, the present study aimed to evaluate palliative care awareness among caregivers of cancer patients and to identify sociodemographic and clinical factors associated with awareness levels.

## 2. Materials and Methods

### 2.1. Study Design and Participants

This study was designed as a descriptive cross-sectional study conducted to evaluate the level of palliative care awareness among caregivers of cancer patients. The study population consisted of caregivers accompanying cancer patients who attended medical oncology clinics for treatment or follow-up during a three-month period.

A total of 550 caregivers were recruited from two oncology centers, including one university hospital and one private hospital. No formal a priori sample size calculation was performed because this study was designed as a descriptive cross-sectional study. Instead, all eligible caregivers who met the inclusion criteria and agreed to participate during the study period were consecutively recruited. Participation in the study was entirely voluntary. Before enrollment, all participants received detailed information regarding the study objectives, procedures, potential risks and benefits, confidentiality, and their right to withdraw at any time without affecting the patient’s medical care. Written informed consent was obtained from all participants prior to data collection.

Caregivers aged 18 years or older who accompanied patients diagnosed with cancer at the oncology outpatient clinics were eligible for inclusion. Individuals younger than 18 years of age, those with cognitive impairment or inability to understand the questionnaire, and individuals accompanying patients without a cancer diagnosis were excluded from the study.

### 2.2. Data Collection

Data were collected using a structured questionnaire developed by the researchers following a comprehensive review of the published literature on palliative care awareness and caregiver experiences. The questionnaire was reviewed by medical oncologists experienced in palliative care to ensure its clarity, relevance, and content validity before implementation. The questionnaire consisted of four main sections:

1. Sociodemographic characteristics (age, gender, education level, marital status, and employment status);
2. Socioeconomic and occupational characteristics;
3. Clinical characteristics of the patients (cancer diagnosis and duration since diagnosis);
4. Awareness and knowledge of palliative care.

The questionnaires were administered through face-to-face interviews conducted by the researchers, with each interview lasting approximately 10–15 min. The primary outcome of this study was palliative care awareness, defined as caregivers’ self-reported familiarity with the concept of palliative care and their perceived level of knowledge. The primary outcome of this study was palliative care awareness, defined as caregivers’ self-reported familiarity with the concept of palliative care and their perceived level of knowledge. Before implementation, the questionnaire was reviewed by experienced medical oncologists to ensure the clarity, relevance, and comprehensiveness of its content. Participants were first asked whether they had previously heard of palliative care. Those who answered “yes” were subsequently asked to rate their level of knowledge as either sufficient or insufficient. Accordingly, awareness was classified into three categories: (1) never heard of palliative care, (2) heard of palliative care but reported insufficient knowledge, and (3) heard of palliative care and reported sufficient knowledge. Because no validated Turkish instrument specifically designed to assess palliative care awareness among caregivers was available, a study-specific questionnaire was used. Although the questionnaire underwent expert review for content validity, formal psychometric validation was not performed.

### 2.3. Ethical Considerations

The study was conducted in accordance with the principles of the Declaration of Helsinki. Ethical approval was obtained from the Institutional Ethics Committee prior to the initiation of the study [Approval No: 2024/201]. All participants were informed about the purpose and scope of the study, and voluntary participation was ensured. Written informed consent was obtained from all participants before the interviews were conducted.

### 2.4. Statistical Analysis

Statistical analyses were performed using IBM SPSS Statistics for Windows, version 32 (IBM Corp., Armonk, NY, USA). Descriptive statistics were used to summarize the characteristics of the participants, including frequencies, percentages, means, and standard deviations.

Associations between categorical variables and palliative care awareness levels were evaluated using the chi-square test. Variables found to be significant in univariate analyses were subsequently included in a multivariable logistic regression model to identify independent predictors of palliative care awareness.

Results of the logistic regression analysis were presented as beta coefficients [β], odds ratios [OR], and 95% confidence intervals [95% CI]. A *p*-value of <0.05 was considered statistically significant.

## 3. Results

### 3.1. Characteristics of the Participants

A total of 550 caregivers of patients with cancer were included in the study. The study population comprised caregivers with diverse sociodemographic characteristics and caregiving relationships. Patients represented a broad spectrum of cancer types and disease stages, with stage IV disease being the most frequent. Approximately half of the participants reported no knowledge of palliative care, whereas the remaining participants reported either sufficient or insufficient knowledge of the concept. Detailed sociodemographic and clinical characteristics of the caregivers are presented in Table 1.

### 3.2. Association Between Sociodemographic and Clinical Factors and Palliative Care Awareness

The associations between sociodemographic and clinical characteristics and palliative care awareness are summarized in Table 2. Age, educational level, employment status, caregiver relationship to the patient, and disease stage were significantly associated with palliative care awareness, whereas gender, marital status, and primary cancer diagnosis were not. Higher awareness levels were observed among older caregivers, participants with higher educational attainment, full-time employed individuals, caregivers who were the patients’ children, and those caring for patients with more advanced disease. To further investigate the association between disease stage and palliative care awareness, an additional analysis compared caregivers of patients with stage IV disease and those caring for patients with stage I–III disease. Caregivers of patients with stage IV disease demonstrated significantly higher levels of palliative care awareness than caregivers of patients with non-stage IV disease (*p* < 0.001).

### 3.3. Experiential Factors Associated with Palliative Care Awareness

The associations between experiential factors and palliative care awareness are presented in Table 3. Source of information, receiving information or recommendations about palliative care, having relatives who had received palliative care, and reasons for not receiving palliative care were significantly associated with awareness levels. In contrast, neither the relationship with the person who had received palliative care nor the time since diagnosis were significantly associated with awareness.

Higher awareness levels were observed among caregivers who obtained information from healthcare professionals, had previously received information or recommendations about palliative care, or had relatives with prior experience of palliative care services. Conversely, caregivers who perceived palliative care as unnecessary demonstrated the lowest awareness levels.

### 3.4. Multivariable Logistic Regression Analysis

A multivariable logistic regression analysis was performed to identify independent predictors of palliative care awareness. Educational level, employment status, age, disease stage, caregiver relationship to the patient, and previous exposure to palliative care were identified as independent predictors of awareness. Among these variables, previous exposure to palliative care and higher educational level showed the strongest associations with increased awareness. Detailed results of the logistic regression analysis are presented in Table 4.

## 4. Discussion

This study evaluated the level of palliative care awareness among caregivers of cancer patients and investigated the sociodemographic and clinical factors associated with this awareness. The findings indicate that awareness of palliative care remains limited among caregivers in Turkey, although several individual and experiential factors significantly influence awareness levels. These results are consistent with previous international studies reporting generally low levels of public awareness regarding palliative care across different countries [1,2,3,4,5,6,7,8,9,10,11,12,13,14,15,16,17,18]. The findings should also be interpreted within the context of the Turkish healthcare system. Although palliative care services have expanded considerably in recent years through the establishment of dedicated palliative care units in hospitals, public awareness and timely utilization of these services remain limited. Consequently, many caregivers may not become familiar with the principles and benefits of palliative care until patients develop advanced disease or complex supportive care needs.

One of the most important findings of this study was the strong association between educational level and palliative care awareness. Caregivers with a university education demonstrated significantly higher awareness compared with those with lower levels of education. This finding is closely related to the concept of health literacy, which refers to individuals’ ability to access, understand, and use health-related information effectively [19]. Higher levels of education are often associated with better access to information sources and improved capacity to interpret medical terminology and healthcare services. Similar findings have been reported in studies conducted in India and China, where higher educational attainment was associated with greater familiarity with the concept of palliative care [20,21]. This association may be particularly relevant in Turkey, where national surveys have demonstrated that a substantial proportion of the adult population has limited or inadequate health literacy. Limited health literacy may reduce individuals’ ability to understand the role of palliative care, distinguish it from end-of-life care, and navigate available healthcare services. Therefore, improving health literacy through public education initiatives and effective communication by healthcare professionals may enhance caregivers’ awareness and facilitate earlier integration of palliative care into routine oncology practice. These results highlight the importance of educational initiatives and public awareness campaigns aimed at improving knowledge of palliative care in the general population.

Age was another factor associated with higher awareness levels in this study. Caregivers aged 45 years and older demonstrated significantly greater awareness compared with younger participants. This finding may be explained by increased exposure to healthcare services, a greater likelihood of experiencing chronic illness within the family, and accumulated life experiences related to disease and caregiving. A study conducted in the United States similarly reported higher levels of palliative care awareness among older caregivers, suggesting that experiential learning may play an important role in shaping awareness [22].

In contrast, no statistically significant difference was found between male and female caregivers in terms of awareness levels, although slightly higher awareness rates were observed among men in the present study. The role of gender in caregiving and awareness of supportive care services remains complex and may vary across cultural contexts. Some studies have suggested that female caregivers may experience greater emotional burden, which could influence their perceptions of palliative care, whereas other studies report that women often demonstrate higher engagement in caregiving roles and healthcare decision-making [23,24]. These mixed findings indicate that gender-related differences in awareness should be interpreted within broader sociocultural frameworks.

Employment status also emerged as a significant factor associated with awareness. Full-time employed caregivers demonstrated the highest awareness levels compared with unemployed or retired individuals. One possible explanation is that individuals who are actively engaged in the workforce may have broader social networks, greater exposure to information sources, and increased interaction with healthcare systems. Similar findings have been reported in studies conducted in Korea, where active employment was associated with higher familiarity with palliative care services [25].

Another important determinant of awareness identified in this study was the relationship between the caregiver and the patient. Caregivers who were the children of the patients demonstrated higher awareness levels compared with spouses or other relatives. This finding may reflect the greater involvement of adult children in medical decision-making processes and coordination of healthcare services. A study conducted in China similarly reported that palliative care awareness was higher among caregivers who were the patients’ children, likely due to their more active participation in care planning and communication with healthcare providers [21].

The stage of the patient’s disease was also significantly associated with caregiver awareness. Caregivers of patients with advanced-stage cancer demonstrated higher levels of awareness compared with those caring for patients with earlier-stage disease. As the disease progresses, symptom burden often increases and the need for supportive care becomes more apparent. In such situations, caregivers may have more frequent interactions with healthcare professionals and multidisciplinary care teams, which may facilitate exposure to information about palliative care services [26,27].

In contrast, the type of cancer diagnosis was not significantly associated with awareness levels. This finding suggests that awareness of palliative care may be influenced more by overall experiences within the healthcare system rather than by specific cancer types. Similar results have been reported in previous studies that found no significant differences in palliative care awareness across different cancer diagnoses [22,28].

Experiential factors also played an important role in shaping awareness. Caregivers who received information about palliative care from healthcare professionals, such as physicians or nurses, demonstrated significantly higher awareness compared with those who obtained information through social networks or media sources. This finding underscores the critical role of healthcare professionals in promoting awareness and understanding of palliative care. Nurses, in particular, often maintain close communication with caregivers and can play a key role in providing education and support related to palliative care services [27,29]. A systematic review conducted in Europe reported that educational interventions delivered by healthcare professionals could increase public awareness of palliative care by up to 30% [30].

Receiving direct information or recommendations regarding palliative care was also associated with higher awareness levels. Caregivers who had previously been informed about palliative care demonstrated significantly greater awareness compared with those who had not received such information. These findings support previous studies indicating that educational interventions, counseling sessions, and informational programs can significantly improve awareness and understanding of palliative care among both patients and caregivers [31,32].

Furthermore, caregivers who had previously encountered individuals receiving palliative care services demonstrated higher levels of awareness. This observation suggests that experiential learning and direct observation of palliative care services may contribute to improved understanding of the concept and benefits of supportive care. Previous studies have also reported that active involvement of family members in palliative care processes may positively influence both knowledge and psychological adaptation among caregivers [27].

Misconceptions about palliative care remain an important barrier to its acceptance. In the present study, caregivers who believed that palliative care was unnecessary or interpreted it as “giving up on life” demonstrated lower awareness levels. These findings reflect a common misconception that equates palliative care with end-of-life care alone. Previous studies have similarly identified the association between palliative care and death as one of the most significant barriers to awareness and utilization of supportive care services [33,34]. These misconceptions may be further reinforced by cultural characteristics in Turkey, where family members traditionally assume a central role in caring for relatives with serious illnesses and are actively involved in treatment-related decision-making. In addition, discussions about prognosis, end-of-life care, and palliative care may be delayed because of cultural sensitivities surrounding serious illness and death. Consequently, palliative care may be perceived as an indication that curative treatment has ended rather than as a supportive approach that can be integrated alongside active cancer treatment.

Interestingly, the duration of the patient’s cancer diagnosis was not significantly associated with awareness levels in this study. This finding suggests that awareness may be shaped more by information access and experiential exposure than by the length of time since diagnosis. Improving palliative care awareness among caregivers may contribute to earlier integration of supportive care services and ultimately improve the quality of life of both patients and families.

## 5. Limitations

This study has several limitations that should be considered when interpreting the findings. First, the cross-sectional design precludes the establishment of causal relationships between the variables examined and palliative care awareness. Second, although participants were recruited from two oncology centers, the findings may not be fully generalizable to all caregivers of patients with cancer in Turkey. Third, the relatively small number of participants in certain subgroups, particularly those defined by relationship to the patient and sources of information, may have limited the statistical power of some analyses. In addition, several potentially important caregiver-related variables, including caregiving duration, caregiving intensity, caregiver burden, and place of residence (urban versus rural), were not collected and therefore could not be evaluated. Furthermore, although the questionnaire was developed based on the available literature and expert review, it was not formally psychometrically validated, which may have influenced the measurement of palliative care awareness. Despite these limitations, the study provides valuable insights into the multidimensional factors influencing palliative care awareness among caregivers of cancer patients in Turkey and contributes to the growing body of literature on this topic. In addition, although participants were recruited from both a university hospital and a private hospital, the recruitment site was not recorded as an analytical variable. Therefore, potential differences between healthcare settings could not be evaluated. Future multicenter studies should investigate whether caregiver awareness differs across healthcare settings.

## 6. Conclusions

The findings of this study indicate that awareness of palliative care among caregivers of cancer patients in Turkey remains limited. Sociodemographic characteristics, access to information, and prior exposure to palliative care services play an important role in shaping awareness levels. Higher educational attainment, active employment, older age, advanced disease stage, and direct experience with palliative care services were identified as key determinants of awareness.

These results highlight the importance of improving public education and communication strategies related to palliative care. Increasing awareness through national educational programs, media campaigns, and structured information provided by healthcare professionals may facilitate earlier integration and broader acceptance of palliative care services. In particular, physicians and nurses have a critical role in informing caregivers and guiding them throughout the care process.

Future research should consider using mixed-method approaches that combine quantitative and qualitative methodologies in order to better understand caregivers’ perceptions, motivations, and barriers related to palliative care awareness.

## Figures and Tables

**Table 1:** Sociodemographic and clinical characteristics.

Variable	Category	*n* (%)
Gender	Female	266 (48.4)
	Male	284 (51.6)
Marital status	Married	474 (86.2)
	Single	76 (13.8)
Education level	Primary school	166 (30.2)
	Middle school	60 (10.9)
	High school	174 (31.6)
	University	150 (27.3)
Employment status	Unemployed	207 (37.6)
	Self employed	109 (19.8)
	Full time	166 (30.2)
	Retired	68 (12.4)
Relationship with patient	Mother	38 (6.9)
	Father	43 (7.8)
	Sibling	50 (9.1)
	Child	216 (39.3)
	Spouse	200 (36.4)
Cancer diagnosis	Lung	109 (19.8)
	Prostate	45 (8.2)
	Breast	167 (30.4)
	Colon	74 (13.5)
	Gastric	54 (9.8)
	Gynecological	56 (10.2)
	Other	45 (8.2)
Disease stage	Stage I	113 (20.5)
	Stage II	120 (21.8)
	Stage III	113 (20.5)
	Stage IV	204 (37.2)
Palliative care awareness	No knowledge	277 (50.4)
	Yes, sufficient knowledge	141 (25.6)
	Yes, but insufficient knowledge	131 (23.8)
Source of information about palliative care	Physician	83 (31.8)
	Nurse	8 (3.1)
	Social network	162 (62.1)
	Media	8 (3.1)
Was palliative care recommended to the patient?	Yes	70 (24.7)
	No	213 (75.3)
Previous exposure to palliative care in relatives	Yes	90 (16.9)
	No	442 (83.1)
Relationship with person receiving palliative care	Grandfather	25 (28.4)
	Grandmother	17 (19.3)
	Sibling	13 (14.8)
	Father	10 (11.4)
	Other	23 (26.1)
Reasons for not receiving palliative care	Ongoing curative treatment	92 (29.2)
	Lack of belief in benefit	48 (15.2)
	Perception of giving up on life	31 (9.8)
	No perceived need	144 (45.8)

**Table 2:** Association Between Sociodemographic and Clinical Variables and Palliative Care Awareness.

Variable	Category	No Knowledge *n* (%)	Yes, Sufficient Knowledge *n* (%)	Yes, But Insufficient Knowledge *n* (%)	Total *n* (%)	*p* Value
Gender	Male	131 (47.1)	77 (27.2)	75 (25.7)	283 (100)	0.120
	Female	146 (54.9)	64 (24.1)	56 (21.0)	266 (100)	
Age group	18–45 years	186 (55.2)	75 (22.3)	76 (22.5)	337 (100)	0.034
	46–65 years	68 (45.3)	51 (34.0)	31 (20.7)	150 (100)	
	>65 years	23 (38.3)	15 (25.0)	22 (36.7)	60 (100)	
Marital status	Married	242 (51.1)	124 (26.2)	107 (22.6)	473 (100)	0.282
	Single	34 (45.9)	17 (23.0)	23 (31.1)	74 (100)	
Education level	İlliterate	12 (80.0)	1 (6.7)	2 (13.3)	15 (100)	<0.001
	Primary school	118 (78.1)	15 (9.9)	18 (11.9)	151 (100)	
	Middle school	32 (53.3)	14 (23.3)	14 (23.3)	60 (100)	
	High school	81 (46.8)	31 (17.9)	61 (35.3)	173 (100)	
	University	34 (22.7)	80 (53.3)	36 (24.0)	150 (100)	
Employment status	Unemployed	132 (63.8)	30 (14.5)	45 (21.7)	207 (100)	<0.001
	Self employed	63 (57.8)	16 (14.7)	30 (27.5)	109 (100)	
	Full time	46 (28.0)	75 (45.7)	43 (26.2)	164 (100)	
	Retired	35 (51.5)	20 (29.4)	13 (19.1)	68 (100)	
Relationship with patient	Spouse	118 (59.3)	36 (18.1)	45 (22.6)	199 (100)	<0.001
	Child	79 (36.6)	82 (38.0)	55 (25.5)	216 (100)	
	Parent	53 (61.6)	11 (12.8)	17 (19.8)	81 (100)	
	Sibling	27 (54.0)	12 (24.0)	11 (22.0)	50 (100)	
Primary cancer diagnosis	Breast	79 (47.3)	42 (25.1)	46 (27.5)	167 (100)	0.248
	Lung	58 (53.2)	26 (23.9)	25 (22.9)	109 (100)	
	Colon	42 (56.8)	17 (23.0)	15 (20.3)	74 (100)	
	Other	98 (56.3)	56 (32.2)	25 (11.5)	179 (100)	
Disease stage	Stage I	62 (55.4)	25 (22.3)	25 (22.3)	112 (100)	<0.001
	Stage II	71 (59.2)	27 (22.5)	22 (18.3)	120 (100)	
	Stage III	71 (62.8)	17 (15.0)	25 (22.1)	113 (100)	
	Stage IV	72 (35.5)	72 (35.5)	59 (29.0)	203 (100)	

**Table 3:** Association Between Experiential Factors and Palliative Care Awareness.

Variable	Category	No Knowledge *n* (%)	Yes, Sufficient Knowledge *n* (%)	Yes, But Insufficient Knowledge *n* (%)	Total *n* (%)	*p* Value
Source of information	Physician	1 (1.2)	57 (68.7)	25 (30.1)	83 (100)	0.004
	Nurse	0 (0.0)	6 (75.0)	2 (25.0)	8 (100)	
	Family/Friends	2 (1.7)	43 (37.4)	70 (60.9)	115 (100)	
	Social media	0 (0.0)	19 (40.4)	28 (59.6)	47 (100)	
	Television/Radio	0 (0.0)	4 (50.0)	4 (50.0)	8 (100)	
Received information or recommendation about palliative care	Yes	1 (1.4)	45 (64.3)	24 (34.3)	70 (100)	0.006
	No	19 (8.8)	91 (42.3)	105 (48.8)	215 (100)	
Having relatives who received palliative care	Yes	4 (4.4)	53 (58.9)	33 (36.7)	90 (100)	<0.001
	No	261 (59.2)	85 (19.3)	95 (21.5)	441 (100)	
Relationship with person receiving palliative care	Grandfather	1 (4.0)	14 (56.0)	10 (40.0)	25 (100)	0.160
	Grandmother	0 (0.0)	9 (52.9)	8 (47.1)	17 (100)	
	Sibling	1 (7.7)	8 (61.5)	4 (30.8)	13 (100)	
	Uncle	0 (0.0)	6 (75.0)	2 (25.0)	8 (100)	
	Aunt	0 (0.0)	0 (0.0)	5 (100.0)	5 (100)	
Time since diagnosis	Ongoing treatment	33 (35.9)	27 (29.3)	32 (34.8)	92 (100)	<0.001
	Lack of belief in benefit	18 (37.5)	16 (33.3)	14 (29.2)	48 (100)	
	Perception of giving up on life	4 (12.9)	16 (51.6)	11 (35.5)	31 (100)	
	No perceived need	116 (80.6)	18 (12.5)	10 (6.9)	144 (100)	
Time since diagnosis	≤1 year	135 (51.3)	72 (27.4)	56 (21.3)	263 (100)	0.485
	1–5 years	102 (47.9)	53 (24.9)	58 (27.2)	213 (100)	
	≥5 years	39 (56.5)	15 (21.7)	15 (21.7)	69 (100)	

**Table 4:** Multivariable Logistic Regression Analysis of Factors Associated with Palliative Care Awareness.

Variable	β	OR	95% CI	*p* Value
Education level	0.72	2.05	1.35–3.15	<0.001
Employment status	0.58	1.79	1.23–2.61	0.003
Age	0.04	1.04	1.01–1.07	0.004
Disease stage	0.29	1.34	1.05–1.72	0.016
Relationship with patient	0.66	1.94	1.18–3.17	0.008
Previous exposure to palliative care	0.85	2.34	1.42–3.84	<0.001

## Data Availability

The data presented in this study are available from the corresponding author upon reasonable request. The data are not publicly available because they contain information that could compromise the privacy of research participants and are subject to ethical restrictions.
